# CD1d-Expressing Breast Cancer Cells Modulate NKT Cell-Mediated Antitumor Immunity in a Murine Model of Breast Cancer Metastasis

**DOI:** 10.1371/journal.pone.0020702

**Published:** 2011-06-13

**Authors:** Laura M. Hix, Yihui H. Shi, Randy R. Brutkiewicz, Paul L. Stein, Chyung-Ru Wang, Ming Zhang

**Affiliations:** 1 Department of Molecular Pharmacology and Biological Chemistry, Northwestern University Feinberg School of Medicine, Chicago, Illinois, United States of America; 2 Department of Microbiology and Immunology, The Walther Oncology Center and Cancer Institute, Indiana University School of Medicine, Indianapolis, Indiana, United States of America; 3 Department of Dermatology, Northwestern University Feinberg School of Medicine, Chicago, Illinois, United States of America; 4 Department of Microbiology and Immunology, Northwestern University Feinberg School of Medicine, Chicago, Illinois, United States of America; Cornell University, United States of America

## Abstract

**Background:**

Tumor tolerance and immune suppression remain formidable obstacles to the efficacy of immunotherapies that harness the immune system to eradicate breast cancer. A novel syngeneic mouse model of breast cancer metastasis was developed in our lab to investigate mechanisms of immune regulation of breast cancer. Comparative analysis of low-metastatic vs. highly metastatic tumor cells isolated from these mice revealed several important genetic alterations related to immune control of cancer, including a significant downregulation of *cd1d1* in the highly metastatic tumor cells. The *cd1d1* gene in mice encodes the MHC class I-like molecule CD1d, which presents glycolipid antigens to a specialized subset of T cells known as natural killer T (NKT) cells. We hypothesize that breast cancer cells, through downregulation of CD1d and subsequent evasion of NKT-mediated antitumor immunity, gain increased potential for metastatic tumor progression.

**Methodology/Principal Findings:**

In this study, we demonstrate in a mouse model of breast cancer metastasis that tumor downregulation of CD1d inhibits iNKT-mediated antitumor immunity and promotes metastatic breast cancer progression in a CD1d-dependent manner *in vitro* and *in vivo.* Using NKT-deficient transgenic mouse models, we demonstrate important differences between type I and type II NKT cells in their ability to regulate antitumor immunity of CD1d-expressing breast tumors.

**Conclusions/Significance:**

The results of this study emphasize the importance of determining the CD1d expression status of the tumor when tailoring NKT-based immunotherapies for the prevention and treatment of metastatic breast cancer.

## Introduction

Significant progress has been made over the past few decades in developing breast cancer immunotherapies that inhibit tumor progression and prevent metastasis [1]. While recent advances in tumor vaccines and T cell-based immunotherapies appear promising, tumor tolerance and immune suppression remain formidable obstacles to eradicating breast cancer [2]. It is widely believed in the field of metastasis that tumor cells must acquire multiple genetic alterations to enable colonization in distant organ sites [3]. Of these, evasion of host immune surveillance is an early and critical step. Cancer cells, like bacteria and viruses, are known to evolve a number of strategies to escape immune surveillance [4], [5]. For example, cancer cells have been documented to downregulate or alter MHC class I molecules and their presentation of tumor antigens to escape immune surveillance, a process known as immunoediting [6], [7]. Identifying critical genetic alterations that enable immune evasion and tumor tolerance will facilitate the development of immunotherapies that eliminate breast cancer.

In order to identify potential gene signatures for metastasis using our syngeneic mouse model of breast cancer metastasis, we compared low-metastatic TM40D breast cancer cells with the highly metastatic TM40D-MB cells by microarray [8]. This revealed a number of immune response genes altered between these cells that were not identified in previous arrays using immune deficient xenograft mouse models [9]. Of these, a significant downregulation was found in the TM40D-MB cells of the *cd1d1* gene, encoding the MHC class I-like molecule CD1d. CD1d molecules present glycolipid antigens to a specialized class of immune cells known as natural killer T (NKT) cells [10]. NKT cells can be divided into two main types: Type I NKT cells, or invariant NKT (iNKT) cells, are characterized by an invariant TCRα chain consisting of Vα14Jα18 gene segments in mice (Vα24Jα18 in humans) and can promote either Th_1_ or Th_2_ effector responses, depending on their activation [11], [12]. Type II NKT cells are a heterogeneous class of CD1d-restricted cells with a diverse TCR repertoire, and have mainly immune regulatory functions [13]. In cancer, accumulated evidence points to a protective role for type I (iNKT) cells, whereas type II NKT cells have been shown to be mainly immunosuppressive [14]. Clinically, iNKT levels are significantly reduced in solid tumors, and low levels of circulating iNKT cells correlate with a poor prognosis in many types of cancers, including breast cancer [15],[16],[17],[18].

Multiple preclinical and clinical studies support the notion that inducing iNKT cell activation can inhibit tumor progression and promote lasting tumor immunity [19], [20]. Activated iNKT cells can rapidly produce pro-inflammatory cytokines such as IFN-γ, which activates innate natural killer (NK) effector function and induces maturation of dendritic (DC) cells that produce immune-stimulating IL-12 [21]. This leads to activation of secondary immune effector responses, including maturation of CD8^+^ T cells to antigen-specific antitumor cytotoxic T lymphocytes (CTL) [22], [23]. In addition to their role in activating antitumor NK and T effector function, iNKT cells can induce direct cytolysis of tumor cells [24]. Activated iNKT cells have been demonstrated to be directly cytotoxic to CD1d-bearing tumor cells in a CD1d-dependent manner *in vitro,* and studies have directly correlated tumor expression of CD1d to their sensitivity to iNKT-mediated antitumor immunity *in vivo* [25],[26].

CD1d is widely expressed in humans and animals in both hematopoietic and non-hematopoietic cells, including multiple tumor types [27], [28]. CD1d downregulation by human papillomavirus (HPV) in infected cervical epithelial cells has been recently shown to be correlated with their progression to cervical carcinoma [29]. Downregulation of CD1d in highly metastatic breast cancer cells may similarly enable evasion of immune surveillance and facilitate metastatic progression. To date, no study has directly linked CD1d expression by breast cancer cells and iNKT-mediated antitumor immunity in preventing breast cancer metastasis.

In this study, we provide the first evidence that in human breast cancer cells, downregulation of CD1d expression is correlated with increasing metastatic potential. Using our syngeneic mouse model of breast cancer metastasis, we show that tumor cells expressing CD1d promote increased iNKT-mediated antitumor immunity in a CD1d-dependent manner *in vitro* and *in vivo.* Importantly, inhibition of tumor CD1d expression *in vivo*, by either antibody blockade or gene silencing, promotes spontaneous breast cancer metastasis. Interestingly, by comparing tumor growth of CD1d-expressing and CD1d-deficient tumor cells in NKT-deficient transgenic knockout (KO) models, we have uncovered important differences in the regulation of these cells by type I and type II NKT cells. These results demonstrate a previously unrecognized role for CD1d-restricted NKT cells in regulating breast cancer metastasis. In addition, these results point to the CD1d expression status of the tumor as being an important determinant in tailoring NKT-based immunotherapies for the prevention and treatment of metastatic breast cancer. Our findings further support research into designing breast cancer immunotherapies that bolster the activation of type I NKT cells, while inhibiting the suppressive functions of type II NKT cells.

## Materials and Methods

### Mice

Inbred BALB/c mice were purchased from Harlan Sprague Dawley Inc. BALB/c mice homozygous deficient in the *cd1d1* gene (CD1d KO) were purchased from the Jackson Laboratory [30]. Recombinase-activating gene 2-deficient (RAG2 KO) BALB/c mice were purchased from Taconic Laboratories [31]. Jα18 KO (iNKT deficient) on the BALB/c background (at least 8 generations backcrossed by Ram Singh, UCLA) mice were provided by Dr. Randy R. Brutkiewicz (Indiana University School of Medicine, Indianapolis, IN) [32]. All mice were housed in a pathogen-free environment at the Northwestern Center for Comparative Medicine (CCM) facility. All experiments were done in accordance with protocols approved by the CCM Committee on Animal Care (CAR) institutional IACUC and in accordance with AAALAC. Female mice approximately 8 weeks old were used for all experiments.

### Breast cancer cell lines

TM40D mammary tumor cells were derived from the FSK4 mammary epithelial cell line established *in vitro* from normal mouse mammary gland [33]. TM40D-MB tumor cells were isolated from bone by antibiotic selection after intracardiac injection of TM40D cells, according to a modified method by Li et al [34]. TM40D tumors have a low potential for spontaneous lung and bone metastasis after orthotopic mammary gland implantation (7.7%), whereas TM40D-MB tumors are metastatic to lung and highly bone metastatic (53.3%) [8]. The TM40D-shCD1d cell line was created by stable lentiviral knockdown of TM40D cells with a short hairpin RNA (shRNA) sequence against murine *cd1d1* (pLKO.1 vector, clone ID 67863, OpenBiosystems). A scrambled shRNA was also used as a control (TM40D-scr). All cells were grown in DMEM/F12 supplemented with L-glutamine, 50 µg/ml streptomycin, 50 U/ml penicillin and 5% heat-inactivated Fetal Bovine Serum (FBS) (all Invitrogen Gibco BRL). All cells were used within two weeks of culture (passages 4–6).

### *In vivo* orthotopic mouse model of breast cancer metastasis

For all tumor experiments, mice were injected bilaterally into the 4^th^ mammary fat pads with 1×10^6^ tumor cells. Tumor volume measurements were taken every three days, and tumor volume was calculated using the formula: length × width**^2^**/2 [35]. Mice were euthanized at the point at which tumors reached the maximum allowable size of 2 cm, following AAALAC guidelines and the rules set by the IACUC. In order to detect spontaneous lung metastasis, tissues were fixed with Bouin's fixative for 24 hrs, then replaced with 70% EtOH [36]. After 48 hrs, tissues were visualized and photographed under a dissecting light microscope with attached camera (Olympus SZX12, Olympus America Inc).

For the *in vivo* anti-CD1d blocking antibody study, 10 mice were implanted with 1×10^6^ tumor cells. At the point tumors were palpable (day 10 post-implantation), 5 mice per group were administered 200 µg i.p. of anti-CD1d (3C11) blocking antibody or vehicle (hybridoma supernatant) control [37]. These injections were repeated at days 17, 24 and 31 post-tumor implantation, and tumor measurements were taken every three days. At day 45, all mice had reached the maximum allowable tumor size and were subsequently euthanized.

### Real-time reverse transcription-polymerase chain reaction (RT-PCR) assay

To validate the microarray results, RNA was harvested from tumor cell lines (Rneasy kit, Qiagen) and RT-PCR was performed on reverse-transcribed cDNA (Roche) using primers for the murine *cd1d1* gene, and L19 amplification was used as a control for relative levels of total RNA, as previously described [38]. For real-time RT-PCR experiments, the SYBR Green assay (Applied Biosystems) was used for detecting products from the isolated complementary DNA samples on a real-time cycler (ABI7900HT, Applied Biosystems). Reactions for each sample were performed in triplicate, and amplified products were visualized on an agarose gel. The level of target gene expression was normalized against glyceraldehyde 3-phosphate dehydrogenase (GAPDH) expression in each sample. For RT-PCR of the human CD1D transcript, the following cell lines were used: 71N, 81N – normal human mammary epithelial cells, 21PT, ZR-75-1 – non-invasive primary tumor, MCF-7 – minimally invasive primary tumor, MDA-MB-468, MDA-MB-231 – pleural effusion, highly metastatic [39],[40]. RNA was harvested (Trizol, Sigma) and reverse-transcribed to make cDNA (Superscript II, Invitrogen). The following primers were used to amplify a 275 bp fragment from the human CD1D gene: 5′-CGC GCA GCG GCG CTC CGC G-3′ located in exon 1, and 5′-GGA CCA AGG CTT CAG AGA G-3′ located in exon 2. Primers for human GAPDH were used as described previously [41].

### Fluorescence-activated cell sorting (FACS) analysis

At maximum tumor size, spleen and tumor were excised and homogenized to obtain single cell suspensions, and erythrocytes were removed as described [42]. To test for immune cell recruitment in spleen and tumor, 2×10^6^ cells from each sample were preincubated with anti-CD16/CD42 (2.4G2, eBioscience) to avoid non-specific binding of antibodies to FcγR [43]. Cells were stained with the following fluorophore-conjugated anti-mouse monoclonal antibodies: anti-CD1d (1B1), anti-TCRβ, anti-CD49b (DX5), anti-CD4, anti-CD8α, anti-CD69, anti-CD45R (all BD Biosciences), and CD1d tetramers loaded with the αGalCer analog PBS-57 (NIH Tetramer Core Facility, Atlanta GA). The percentage of live cells was assessed using the LIVE/DEAD fixable violet blue cell kit (Invitrogen). Cells were sorted using a FACS Canto II (BD Biosciences) and analyzed on FlowJo software (Tree Star).

### Enrichment of iNKT cells and in vitro cytotoxicity assay

iNKT cells were enriched from splenocytes of healthy, unchallenged 7 week-old wildtype BALB/c by positive selection as previously described [42]. Dead cells were removed from splenocytes using a Dead Cell Removal Kit (MACS, Miltenyi Biotec). To debulk splenocytes of B cells, splenocytes were incubated with biotin-B220 (BD Biosciences), followed by streptavidin microbead magnetic column depletion (MACS, Miltenyi Biotec). For positive selection of iNKT cells, splenocytes were incubated with a PE-conjugated PBS-57-loaded CD1d tetramer, followed by anti-PE magnetic column purification (MACS, Miltenyi Biotec). Percent enrichment and activation status (CD69^+^) of iNKT cells isolated from splenocytes was confirmed by FACS. Activated iNKT-enriched splenocytes were immediately incubated with target tumor cells at effector to target (E:T) ratios of 5∶1, 10∶1 and 25∶1, in the presence or absence of anti-CD1d (3C11, BD Biosciences), or IgM isotype control (Sigma) [44]. After 4 hr incubation at 37°C and 5% CO_2_, cell-free supernatants were harvested and assayed for lactate dehydrogenase (LDH) activity as a measure of cell lysis (LDH Cytotoxicity Detection Kit, Roche) [45]. Spontaneous release of LDH by effector and target cells were controlled by separate incubations of these populations. The results were calculated as follows:

% Cytotoxicity  =  test release – non-specific release x 100

total LDH release – non-specific release

### Statistical analysis

Results are expressed as the median and range and mean ± standard deviation. Student's paired *t* tests or one-way ANOVA were used to determine statistical significance. A value of *P*<0.05 was considered statistically significant. Data were analyzed using Excel for Mac 2008 (Microsoft) and Prism 5 (GraphPad).

## Results

### CD1d expression is correlated with increasing metastatic potential in murine and human breast cancer cells

**Figure 1:**
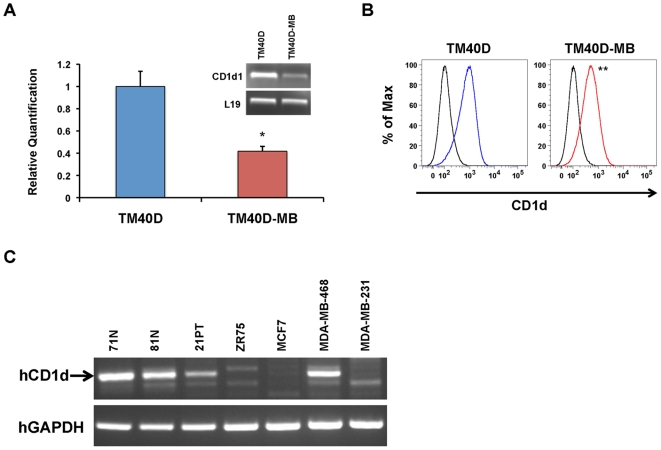
Decreased expression of CD1d in highly metastatic murine and human breast cancer cells. (A) Real-time RT-PCR assay confirming significant downregulation of the CD1d1 gene in TM40D-MB cells, as compared to parental TM40D (low metastatic) cells. L19 serves as an internal control. Experiments were performed in triplicate, and data are represented as the mean ± SEM, * *P*≤0.05. (B) Flow cytometry analysis of CD1d using a PE-conjugated anti-CD1d mAb (1B1) or isotype IgG2b control. Average ± SD mean fluorescence intensity (MFI) for three independent experiments: TM40D (blue)  = 928.7±21.2, TM40D-MB (red)  = 541.0±57, ** *P*≤0.001. (C) Decreased expression of CD1d in human mammary epithelial cells correlates with increasing metastatic potential by RT-PCR. 71N, 81N: normal transformed human mammary epithelial cells. 21PT, ZR75: primary breast adenocarcinoma cells. MCF-7: minimally invasive adenocarcinoma cells. MDA-MB-468, MDA-MB-231: highly metastatic human breast adenocarcinoma cells, from patients of African-American (MDA-MB-468) and Caucasian (MDA-MB-231) descent. GAPDH serves as an internal housekeeping gene control.

Several lines of evidence support the idea that a small population of primary tumor cells possess existing molecular signatures for metastasis [46], [47]. In order to identify potential metastatic signatures using our mouse model of breast cancer metastasis, TM40D breast cancer cells of low metastatic potential were compared to the highly metastatic TM40D-MB cells by microarray [8]. Among the genes that were found differentially expressed between these two cell lines, we found a 4.72-fold downregulation of the *cd1d1* gene in the TM40D-MB cells. (Figure S1). This expression difference was validated by real-time RT-PCR, comparing amplified cDNA for the murine *cd1d1* gene by RT-PCR between the TM40D and TM40D-MB cells (Fig. 1A). Downregulation of surface CD1d expression in the TM40D-MB cells was also confirmed by fluorescence-activated cell sorting (FACS) (Fig. 1B). CD1d is known to be expressed in human breast tissue, in both ductal epithelial and vascular smooth muscle cells [48]. Downregulated CD1d expression has been correlated with decreased iNKT-mediated antitumor immunity in several human and murine hematopoietic malignancies [24], [25], [49]. In human solid tumors, a correlation of downregulated CD1d expression with increasing malignancy has been reported in malignant glioma, and most recently HPV-transformed cervical carcinoma cells [29], [50]. In order to assess the importance of CD1d downregulation in human breast cancer, we analyzed a panel of human mammary epithelial cell lines of increasing metastatic potential by RT-PCR for their expression of CD1d (Fig. 1C). CD1d was found to be expressed in normal human mammary epithelial cells, and with the exception of MDA-MB-468, is downregulated in the transition from normal to malignant breast cancer. These results suggest that downregulation of CD1d expression in both murine and human breast cancer may be an important mechanism for evading tumor immune surveillance and promoting metastatic cancer progression.

### CD1d-expressing tumor cells promote direct iNKT-mediated cytotoxicity in a CD1d-dependent manner *in vitro*

**Figure 2:**
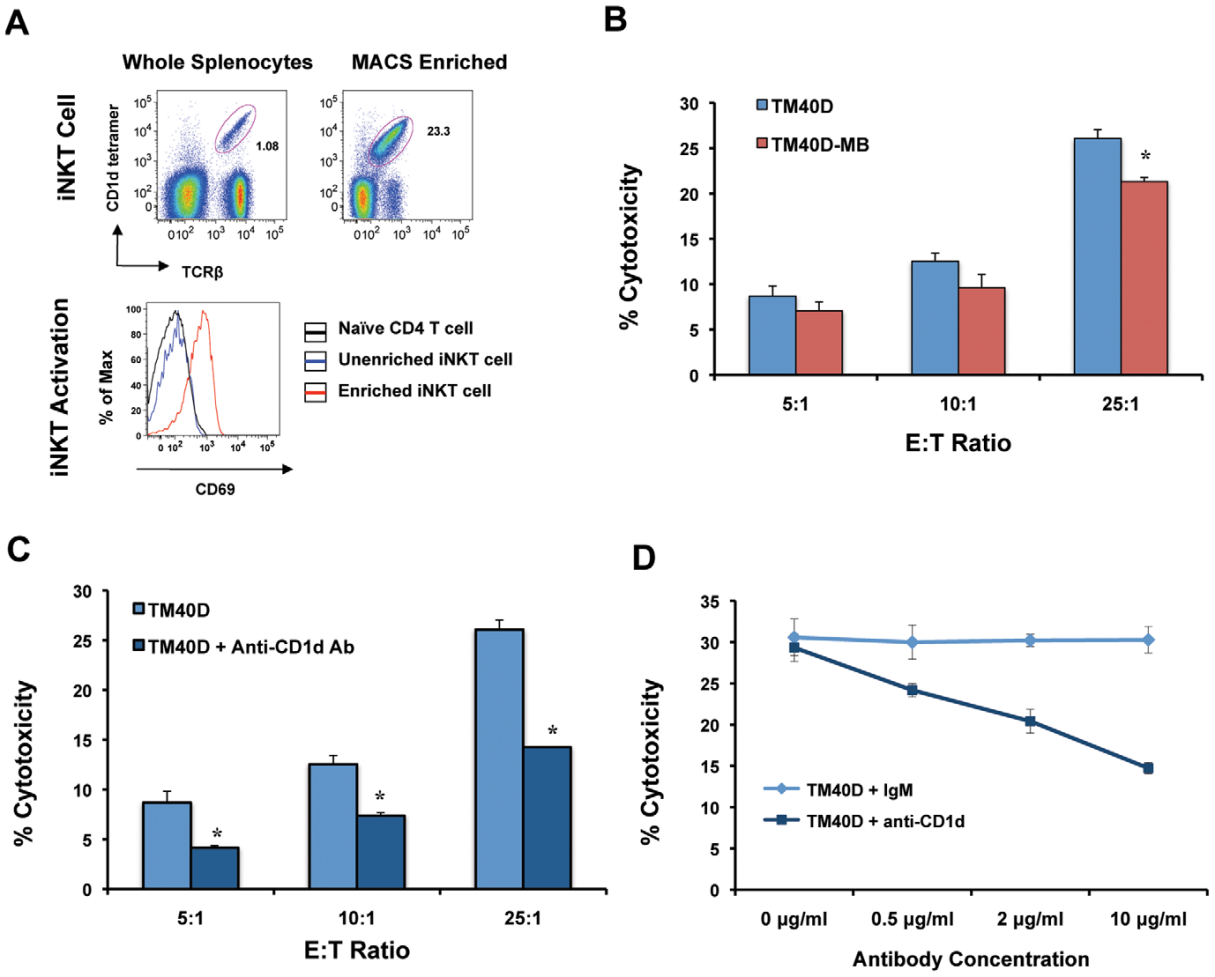
Increased tumor cytolysis of CD1d-expressing cells by enriched iNKT cells. (A) FACS analysis of enriched iNKT cells by magnetic bead sorting using PE-conjugated PBS-57-loaded CD1d tetramer and FITC-conjugated anti-TCRβ Ab. Activation assessed using PerCP-conjugated anti-CD69 Ab. Mean fluorescence intensity (MFI) for control naïve CD4^+^ T Cell = 99 (black), Unenriched iNKT = 121 (blue), Enriched iNKT = 715 (red). (B) *In vitro* Lactate Dehydrogenase (LDH) cytotoxicity assay. Positively-enriched iNKT effector cells (>20% iNKT+) were incubated with TM40D or TM40D-MB target cells at E:T ratios of 5∶1, 10∶1 and 25∶1, for 4 hrs at 37°C, 5% CO_2_. Cell-free supernatants were assayed for LDH activity as a measure of cell lysis. (C) TM40D target cells were incubated with enriched iNKT cells at E:T ratios of 5∶1, 10∶1 and 25∶1, in the presence or absence of anti-CD1d (3C11) blocking antibody (10 µg/ml). (D) *In vitro* anti-CD1d antibody titration in TM40D cells. Positively-enriched iNKT effector cells (>20% iNKT^+^) were incubated with TM40D target cells at an E:T ratio of 25∶1, in the presence of anti-CD1d (3C11) blocking antibody or IgM isotype control, at the concentrations indicated. Data are presented as mean ± SD, * *P*<0.05. Data are representative of at least two independent experiments.

Previous studies in hematopoietic tumors demonstrated the ability of CD1d-expressing tumors to be susceptible to direct iNKT-mediated cytolysis in a CD1d-dependent manner [25], [26], [51]. However, the ability of iNKT cells to induce direct cytolysis of CD1d-expressing breast cancer cells has not been reported. For this experiment, activated iNKT effector cells were enriched from splenocytes by positive selection using a ligand-conjugated CD1d tetramer [42] (Fig. 2A). Activation of iNKT cells by tetramer ligation was positively confirmed by increased expression of CD69, a marker for iNKT activation [52]. Enriched iNKT cells were immediately incubated with either TM40D (CD1d-hi) or TM40D-MB (CD1d-lo) tumor target cells and assayed for tumor cytolysis. TM40D (CD1d-hi) tumor cells demonstrated increased cytotoxicity over TM40D-MB (CD1d-lo) tumor cells in a range of effector to target cell ratios, and significantly higher cytotoxicity at an E:T ratio of 25∶1 (*P*<0.05), as measured by the release of lactate dehydrogenase (LDH) (Fig. 2B). This suggests that enriched iNKT cells may preferentially target tumor cells expressing higher levels of CD1d, and downregulation of CD1d may be a mechanism for evading direct iNKT-mediated cytotoxicity. Additionally, we show that direct iNKT cytolysis of TM40D (CD1d-hi) cells could be partially blocked by the addition of an anti-CD1d blocking antibody (3C11) in a range of effector to target cell ratios (Fig. 2C). The ability of the anti-CD1d antibody to block tumor cytolysis was concentration-dependent, as compared to isotype control (Fig. 2D). These results are consistent with previous reports demonstrating susceptibility of CD1d-expressing hematopoietic tumor cells to direct iNKT-mediated cytolysis, and the ability of anti-CD1d blocking antibody to partially abrogate this effect [26], [51]. To our knowledge, this is the first study to demonstrate the ability of enriched iNKT cells to induce direct cytolysis of CD1d-expressing breast cancer cells in a CD1d-dependent manner *in vitro*.

### Downregulation of CD1d by tumor correlates with evidence of decreased iNKT-mediated antitumor immunity *in vivo*

**Figure 3:**
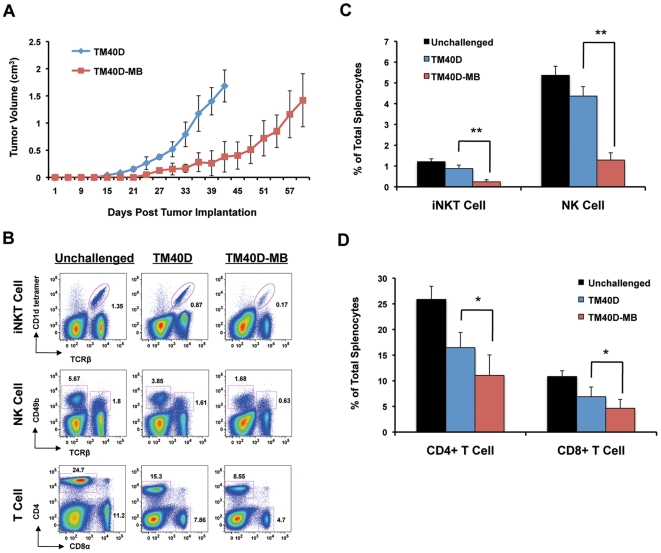
Decreased CD1d expression by tumor correlates with decreased iNKT-mediated antitumor immunity *in vivo*. (A) Orthotopic injection into the bilateral mammary fat pads of mice with either 1×10^6^ CD1d-expressing TM40D or CD1d-deficient TM40D-MB cells, 5 mice per tumor group. Mice were monitored for tumor formation (tumor size of 0.3 cm), and mice in each tumor group were euthanized at maximum tumor size (2 cm). (B) FACS analysis comparing spleens isolated from either TM40D or TM40D-MB tumor-implanted mice. Splenocytes were isolated from mice at maximum tumor size and analyzed by FACS for live iNKT cell populations using APC-conjugated PBS-57-loaded CD1d tetramer and FITC-conjugated anti-TCRβ antibodies. Live NK populations were assessed using PE-conjugated anti-CD49b (DX5) and FITC-conjugated anti-TCRβ antibodies. Live CD4^+^ and CD8^+^ T cell populations were assessed using APC-conjugated anti-CD4 and PerCP-Cy5.5-conjugated anti-CD8α antibodies. *C,*(D) Histograms quantifying the total percentage of live iNKT, NK, CD4^+^ and CD8^+^ T cell of total splenocytes. N = 3 (Unchallenged). N = 5 (TM40D, TM40D-MB). Data are mean ± SD. * *P*≤0.05, ** *P*≤0.001. These results are representative of at least two independent experiments.

The ability of CD1d-restricted iNKT cells to promote antitumor immune responses has been documented in multiple human and animal cancer studies [14], [19], [20]. Several studies have demonstrated a direct role for CD1d-expressing tumor cells in activating iNKT-mediated antitumor immunity [24], [25]. Activated iNKT cells have been shown to prime innate NK immune responses, as well as activate secondary immune effector antitumor CD4^+^ and CD8^+^ T cells [22], [23]. We hypothesize that downregulation of CD1d in breast cancer cells may inhibit iNKT-regulated primary and secondary immune responses. In order to assess the effects of tumor downregulation of CD1d on iNKT-mediated antitumor immunity *in vivo*, we utilized our mouse model of breast cancer metastasis to compare the immune responses of mice implanted with either low metastatic TM40D (CD1d-hi) cells, or highly metastatic TM40D-MB (CD1d-lo) cells. For this experiment, TM40D or TM40D-MB cells were implanted into wildtype BALB/c and monitored for tumor progression. TM40D-MB tumor cells with downregulated CD1d expression were found to grow at a slower rate than TM40D (CD1d-hi) tumors, suggesting that downregulated CD1d expression may not affect tumor proliferation directly (Fig. 3A). In order to assess potential effects of downregulated CD1d expression by tumor on innate and adaptive antitumor immunity, we assessed spleens from tumor-implanted mice for levels of iNKT, NK, CD4^+^ and CD8^+^ T cells by FACS. As a control, these cell populations were compared to spleen from healthy (unchallenged) mice. Splenocytes isolated from the TM40D (CD1d-hi) group demonstrated lower levels of NK and iNKT cells as compared to healthy unchallenged mice, as expected from immune suppression at this late stage of tumor progression (Fig. 3B,C). Importantly, splenic levels of NK and iNKT cells of the TM40D mice were significantly higher than in the TM40D-MB (CD1d-lo) group (NK and iNKT *P*<0.001), although tumor sizes were comparable at time of analysis. This suggests that downregulation of tumor CD1d expression may have an effect on NK and iNKT antitumor immunity, even at a late stage in tumor progression. Both the TM40D and TM40D-MB tumor groups had decreased splenic levels of CD4^+^ and CD8^+^ T cell populations as compared to unchallenged mice, although the reduction in the TM40D-MB group was significantly more pronounced (CD4^+^ *P* = 0.003, CD8^+^ *P* = 0.012) (Fig. 3B,D). These results suggest that secondary T cell-mediated adaptive antitumor immunity may also be compromised by downregulated CD1d expression. In summary, these data point to a correlation between downregulated CD1d expression by tumor and suppression of iNKT-regulated antitumor immunity *in vivo*.

### *In vivo* antibody blockade of CD1d in mice bearing CD1d-expressing tumors promotes spontaneous breast cancer metastasis

**Figure 4:**
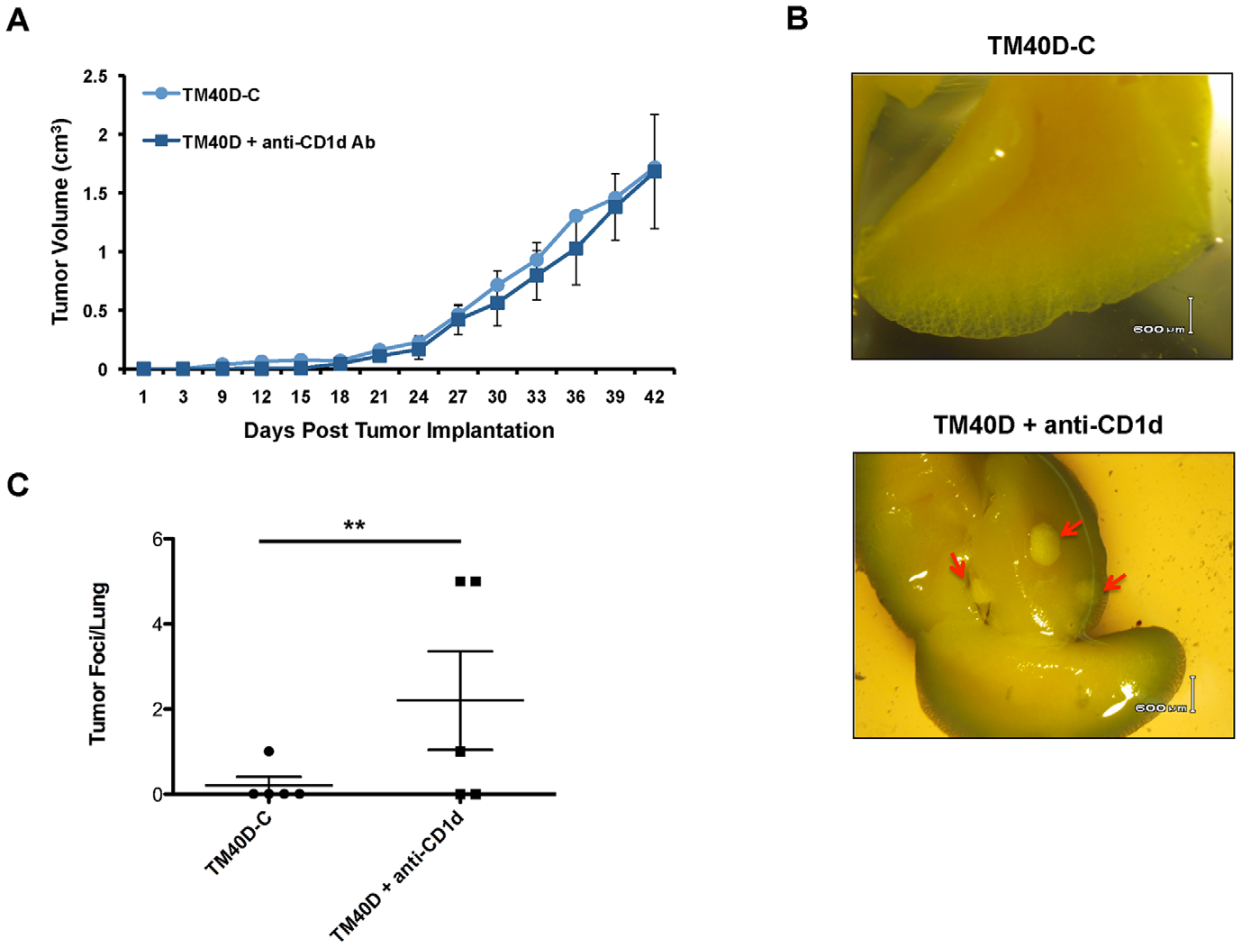
Antibody blocking of CD1d-expressing TM40D tumor cells increases spontaneous lung metastasis *in vivo*. (A) Comparison of tumor growth in mice administered anti-CD1d blocking antibody or vehicle control. Wild-type BALB/c mice (5 mice per group) were implanted with TM40D tumor cells and inoculated I.P. with either with 200 µg of anti-CD1d (3C11) blocking antibody or vehicle control (TM40D-C) at days 10, 17, 24, and 31 post tumor implantation. Mice were monitored for tumor formation (tumor size of 0.3 cm), and mice were euthanized at maximum tumor size (2 cm). (B) *In vivo* anti-CD1d antibody blockade of CD1d-expressing TM40D tumors increases the frequency of lung tumor metastases in mice. At maximum tumor volume, lung tissues were isolated and fixed in Bouin's Fixative and scored for visible metastasis foci under dissecting light microscope. (C) Scatter plot depicting average number of tumor foci counted per lung (** *P*<0.005, one-way ANOVA test).

Previous studies have demonstrated the ability of *in vivo* anti-CD1d antibody blockade to inhibit tumor progression of CD1d-deficient tumors [37], [53], [54]. However, the efficacy of this treatment strategy for CD1d-expressing breast tumors has not been verified. Based on our *in vitro* findings, we hypothesized that *in vivo* blockade of CD1d using an anti-CD1d blocking antibody would inhibit iNKT-mediated antitumor immunity and result in increased breast cancer metastasis. To test this, wildtype BALB/c mice were implanted with 1×10^6^ TM40D tumor cells. At the point at which tumors were palpable (day 10 post tumor implantation), mice were injected intraperitoneally (i.p) with either anti-CD1d (3C11) blocking antibody, or vehicle control [37]. Unlike the anti-CD1d (1B1) blocking antibody used by the Smyth group, anti-CD1d (3C11) is a non-depleting blocking antibody that is not known to activate antitumor APCs [55]. Antibody injections were repeated weekly, and mice were euthanized at maximum tumor volume. Unlike previous studies using antibody blockade of CD1d-deficient tumors, treatment of CD1d-expressing TM40D cells with anti-CD1d blocking antibody did not inhibit tumor growth (Fig. 4A). In order to assess the effect of CD1d antibody blocking on tumor metastasis, we evaluated whether anti-CD1d antibody blockade could increase tumor metastasis to lung. Previous studies in our lab demonstrated that TM40D tumors have a low rate of spontaneous metastasis [35]. As assessed by histology, tumors in mice treated with the anti-CD1d antibody exhibited increased ability to metastasize to lung, with significantly increased numbers of tumor foci per lung (*P* = 0.0049) (Fig. 4B,C). Thus, unlike other CD1d-deficient tumors, treatment of CD1d-expressing breast tumors with an anti-CD1d blocking antibody significantly increases spontaneous tumor metastasis *in vivo*, demonstrating the importance of CD1d expression for iNKT-mediated antitumor immunity.

### Knockdown of CD1d gene expression in TM40D cells promotes increased breast cancer metastasis

**Figure 5:**
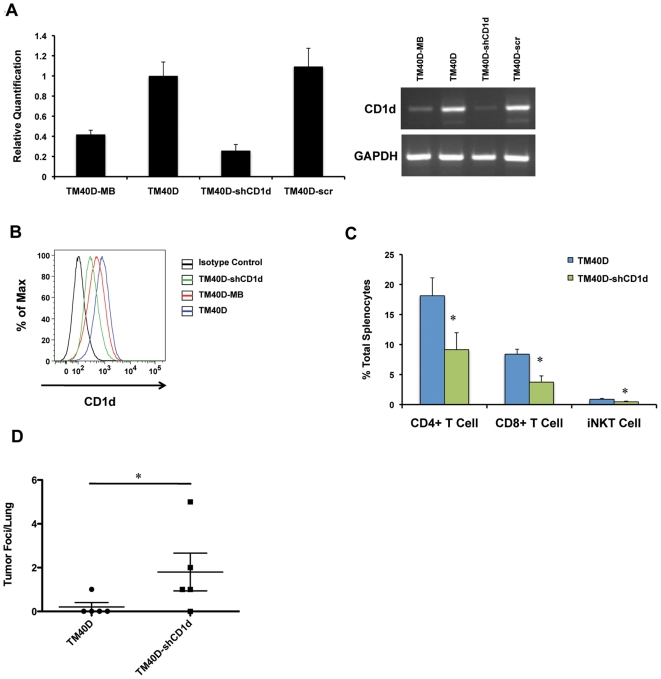
Gene knockdown of CD1d by shRNA in CD1d-expressing TM40D cells suppresses *in vivo* iNKT-mediated antitumor immunity and promotes increased spontaneous metastasis to lung. (A) TM40D cells were transduced by a pLKO.1 lentivirus expressing shRNA against murine *cd1d1*. Real-time RT-PCR assay confirming downregulation of the *cd1d* gene in the TM40D-shCD1d cells, as compared to parental TM40D and TM40D-MB cells and scrambled shRNA control (TM40D-scr). TM40D-MB cells, as compared to parental TM40D (low metastatic) cells. GAPDH serves as an internal control. (B) FACS analysis of gene knockdown of CD1d using a PE-conjugated anti-CD1d mAb (1B1) or isotype IgG2b control. Mean fluorescence intensity (MFI) for IgG2b Isotype Control (black)  = 127, TM40D-shCD1d (green)  = 389, TM40D-MB (red)  = 599, TM40D (blue)  = 932. (C) Suppression of iNKT-regulated lymphocytes *in vivo* of TM40D-shCD1d tumor-implanted mice, as compared to parental TM40D control. Histograms quantifying the total percentage of live iNKT, and CD4^+^ and CD8^+^ T cell of total splenocytes. N = 5 (TM40D, TM40D-shCD1d). Data are mean ± SD. * *P*≤0.05. (D) Scatter plot depicting increased number of tumor foci counted per lung in TM40D-shCD1d tumor-implanted mice as compared to TM40D parental control. (* *P*≤0.05, one-way ANOVA test).

Next we sought to determine the role of tumor-specific CD1d downregulation, in contrast to systemic CD1d inhibition by antibody blockade, in promoting spontaneous breast cancer metastasis. For this experiment, we employed lentiviral delivery of shRNA against murine CD1d (TM40D-shCD1d). Knockdown of CD1d expression level in TM40D cells was verified by real-time RT-PCR, which demonstrated CD1d expression levels in TM40D-shCD1d cells to be downregulated to a level comparable to TM40D-MB (Fig. 5A). FACS analysis of TM40D-shCD1d surface expression of CD1d demonstrated a similar decrease, as compared to TM40D and TM40D-MB cells (Fig. 5B). When TM40D-shCD1d cells were implanted into BALB/c mice, primary tumor growth rates were found to be identical to the parental TM40D-implanted mice (data not shown). Importantly, when spleens were harvested from these mice and assessed for levels of iNKT-mediated immune populations, TM40D-shCD1d mice demonstrated significantly decreased splenic levels of CD4^+^ and CD8^+^ T cells (CD4^+^ *P* = 0.017, CD8^+^ *P* = 0.034), as well as significantly decreased iNKT cells (*P* = 0.003), as compared to parental TM40D-implanted mice (Fig. 5C). NK levels were not significantly affected (data not shown). This supports the notion that downregulation of tumor CD1d expression may play a role in suppressing the antitumor immune functions of iNKT cells. In addition, we analyzed these groups for evidence of lung metastasis, and found a significantly increased rate and frequency of lung foci per lung in the TM40D-shCD1d (*P = *0.0152) mice, as compared to parental TM40D-implanted mice (Fig. 5D). These results demonstrate the importance of tumor-specific CD1d expression for inhibiting metastatic breast cancer progression, and point to the importance of iNKT-mediated antitumor immunity in regulating these tumors.

### Differential regulation of tumor growth and metastasis of CD1d-hi vs. CD1d-lo tumors by CD1d-restricted NKT cells

Recent studies have begun to elucidate a novel immunoregulatory axis of CD1d-restricted NKT cells, with type I invariant NKT (iNKT) having antitumor functions, and type II variant NKT cells demonstrating mainly immunosuppressive tumor-promoting abilities [14]. The role of CD1d-restricted type I vs. type II NKT cells in breast cancer has only been addressed using the CD1d-deficient 4T1 mammary carcinoma model [37]. The ability of CD1d-restricted NKT cells to regulate tumor growth and metastasis of CD1d-expressing breast tumors has yet to be explored. To address this, we compared tumor growth and metastasis rates of TM40D (CD1d-hi) or TM40D-MB (CD1d-lo) cells implanted in wildtype mice, CD1d KO mice that are deficient in all CD1d-restricted NKT cells, and Jα18 KO mice that are deficient in only type I NKT (iNKT) cells [30], [32]. Additionally, we sought to determine the importance of adaptive immunity in regulating tumor growth and immune responses between these tumors using recombinase-activating gene 2 deficient (RAG2 KO) mice, lacking all B and T lymphocytes, including NKT cells [31].

**Figure 6:**
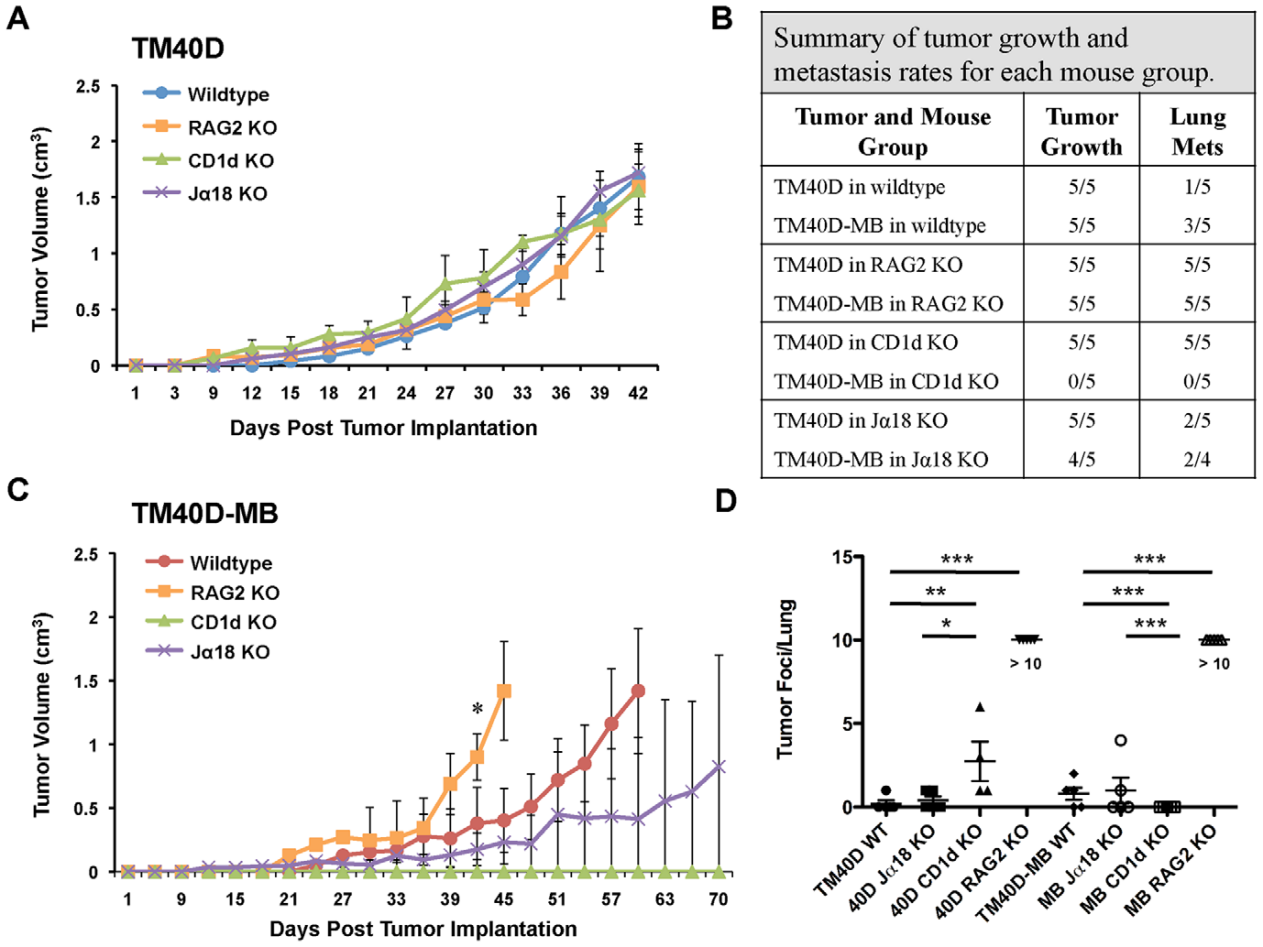
Differences in tumor growth and metastasis between TM40D and TM40D-MB tumors in normal and immune deficient mice. Orthotopic injection into the bilateral mammary fat pads of CD1d KO, Jα18 KO, RAG2 KO, or wildtype BALB/c mice, with either 1×10^6^ TM40D (CD1d-hi) or TM40D-MB (CD1d-lo) cells, 5 mice per tumor group. Mice were monitored for tumor formation (tumor size of 0.3 cm), and mice in each tumor group were euthanized at maximum allowable size (2 cm). (A) Comparison of TM40D (CD1d-hi) tumor growth in CD1d KO, Jα18 KO, RAG2 KO, or wildtype BALB/c mice. (B) Differences in rates of tumor growth and metastasis between TM40D and TM40D-MB tumors in normal and immune deficient mice. (C) Comparison of TM40D-MB (CD1d-lo) tumor growth in CD1d KO, Jα18 KO, RAG2 KO, or wildtype BALB/c mice. Data are mean ± SD. (D) Scatter plot depicting increased number of tumor foci counted per lung in TM40D or TM40D-MB tumor-implanted mice in immune-deficient CD1d KO, Jα18 KO, RAG2 KO, or wildtype BALB/c mice. (* P≤0.05, ** P<0.005, *** P≤0.001, one-way ANOVA test). Data are representative of two independent experiments.

For these experiments, we first compared rates of tumor growth and metastasis of TM40D (CD1d-hi) cells implanted in wildtype, CD1d KO, Jα18 KO and RAG2 KO mice. Tumor growth rates were found to be similar between wildtype and immune-deficient mouse groups (Fig. 6A). These results are in line with previous observations in the CD1d-deficient 4T1 breast cancer mouse model [37]. In contrast to rates of tumor growth, significant differences were found between these mouse groups in their ability to spontaneously metastasize to lung (Fig. 6B,D). TM40D cells were most metastatic in RAG2 KO mice, lacking adaptive immune NKT, T and B lymphocytes, but maintaining NK cell innate immune functions. These results suggest that innate NK-mediated antitumor immunity is not sufficient to inhibit spontaneous metastasis, and adaptive immune lymphocytes are the primary effector cells in our model. Next we addressed the role of CD1d-restricted NKT cells in preventing metastasis. As compared to TM40D in wildtype, the rate of metastasis and overall number of metastases per lung in TM40D-implanted CD1d KO mice was significantly higher (*P = *0.0077). These results demonstrate the importance of CD1d-restricted NKT cells in regulating metastatic progression of CD1d-expressing tumors. We predicted that TM40D tumors implanted in type I NKT-deficient Jα18 KO mice would show similar rates of metastasis as compared to CD1d KO mice, owing to the importance of type I NKT cells in antitumor immunity. Surprisingly, we found a more significant increase in the rate and frequency of lung metastases in TM40D-implanted CD1d KO mice than in the Jα18 KO mice (*P = *0.0164). These results establish the importance of adaptive immunity and CD1d-restricted NKT cells in preventing metastatic cancer progression of CD1d-expressing tumors, but do not directly implicate the importance of type I NKT cells in this model.

In order to determine the importance of CD1d-restricted NKT cells in regulating the immunity of tumors with downregulated CD1d, TM40D-MB (CD1d-lo) tumors were also implanted in wildtype, CD1d KO, Jα18 KO and RAG2 KO mice. In contrast to TM40D tumor growth in these mice, growth of TM40D-MB (CD1d-lo) cells varied dramatically between mouse groups (Fig. 6C). Unlike the TM40D tumors that readily grew in CD1d KO mice, TM40D-MB tumors did not grow at all in these mice (Fig. 6B,C). These results were similar to those observed in CD1d-deficient tumor models (15-12RM, CT26-L5), where CD1d KO mice are highly resistant to tumor growth [37]. This suggests that the presence of suppressive type II NKT cells in these tumors may be more important in promoting tumor progression than the absence of type I NKT cells. Accordingly, we would expect TM40D-MB tumors to be able to grow and metastasize in Jα18 KO mice lacking only type I NKT cells. As predicted, TM40D-MB tumors grew in Jα18 KO mice, although tumor growth rates were on average slower than in wildtype mice (Fig. 6C). Interestingly, in RAG2 KO mice deficient in all adaptive lymphocytes, including NKT cells, TM40D-MB tumor growth rates were significantly increased as compared to wildtype (*P = *0.0127 at day 42 post tumor implantation), and similar to the rate of TM40D in wildtype. These results are in line with previous studies demonstrating increased rates of tumor growth in RAG2 KO mice, owing to the importance of adaptive antitumor immunity [56].

Next we assessed the ability of TM40D-MB (CD1d-lo) tumors to metastasize to lung in these mice, as compared to TM40D (CD1d-hi). While no tumor growth or metastasis was detected in CD1d KO mice implanted with TM40D-MB, Jα18 KO mice demonstrated a similar ability to metastasize to lung as compared to wildtype, with increased number and size of lung tumor foci, although this was not statistically significant (*P* = 0.1877) (Fig. 6B,D). Similar to TM40D, all of the RAG2 KO mice implanted with TM40D-MB tumor cells readily metastasized to lung, and the overall number of lung metastases for both groups were significantly greater than in wildtype mice (*P*<0.001) (Fig. 6B,D). These findings further implicate the role of adaptive immune lymphocytes for preventing breast cancer metastasis in our tumor model. The results of these experiments point to potential differences in the ability of type I and type II NKT cells to regulate antitumor immunity in CD1d-expressing vs. CD1d-deficient tumors.

## Discussion

The ability of CD1d-restricted iNKT cells to promote antitumor immune responses has been documented in multiple human and animal cancer studies [14], [19], [20]. Several tumor types have been shown to express CD1d, and tumor expression of CD1d has been directly correlated with the ability of iNKT cells to induce direct tumor cytolysis *in vitro* and promote iNKT-mediated tumor immunity *in vivo* [25], [51]. To our knowledge, this is the first study to address the role of CD1d expression and NKT-mediated antitumor immunity in regulating breast cancer metastasis. In our mouse model of breast cancer metastasis, we detected a significant downregulation of CD1d in the highly metastatic TM40D-MB cells, as compared to the low-metastatic TM40D cells. We hypothesized that tumors may acquire a selective advantage by downregulating expression of CD1d, thereby evading iNKT-mediated immune surveillance and promoting metastatic cancer progression.

The exact mechanisms of iNKT-mediated antitumor immunity have yet to be fully elucidated, however iNKTs are known to have both direct and indirect effector functions, including the ability to activate both innate NK and adaptive T cell-mediated antitumor immunity [22], [23], [24]. In our study, we show that downregulation of CD1d in highly metastatic cells correlates with *in vivo* suppression of iNKT-regulated immune effector cells, as evidenced by significantly decreased *in vivo* levels of iNKT, NK, CD4^+^ and CD8^+^ T cells. The effect on iNKT immune suppression was not attributed to differences in tumor burden, as all mice were euthanized at the same tumor volume. In addition, while gene knockdown of CD1d in TM40D cells had no effect on primary tumor growth, mice implanted with these tumors demonstrated significantly decreased splenic levels of iNKT and T effector cells and a significant increase in spontaneous lung metastasis. These results point to the importance of CD1d expression by tumor in promoting iNKT-mediated antitumor immunity.

In addition to their indirect antitumor immune functions, multiple studies confirm the ability of iNKTs to be directly cytotoxic to tumor cells in a CD1d-dependent manner [24], [25], [26]. In this study, we demonstrated that enriched iNKT cells are preferentially cytotoxic to breast cancer cells with increased CD1d expression *in vitro*, and this effect could be partially abrogated by the addition of anti-CD1d blocking antibody. Previous studies by the Smyth group reported potent tumor inhibition by administering an anti-CD1d blocking antibody in mice bearing multiple types of CD1d-deficient tumors, presumably by blocking the suppressive functions of type II NKT cells [37], [53], [54]. As these studies were conducted using CD1d-deficient tumors, it was unknown whether similar effects could be achieved with CD1d-expressing tumors. As demonstrated in our study, treatment of mice bearing CD1d-expressing breast tumors *in vivo* with an anti-CD1d blocking antibody did not inhibit tumor progression, but instead significantly increased spontaneous lung metastasis. The importance of CD1d expression by tumor was further validated by shRNA knockdown of CD1d, which also demonstrated significantly increased tumor metastasis to lung. These results highlight a previously unrecognized role for tumor CD1d expression in preventing spontaneous breast cancer metastasis, and provide further evidence to support the direct cytotoxic effector role of iNKT cells in antitumor immunity.

Previous studies in a MCA-induced fibrosarcoma model reported only a minor contribution of direct iNKT antitumor cytotoxicity *in vivo* [57]. In our study, we did not see direct evidence of iNKT cytotoxicity *in vivo* as evidenced by the ability of iNKT cells to affect tumor growth rate. Our highly metastatic TM40D-MB (CD1d-lo) tumor cells actually grow slower than TM40D (CD1d-hi) cells, both *in vitro* (unpublished data) and *in vivo*, and modulation of tumor CD1d expression by either antibody blockade or gene knockdown had no effect on primary tumor growth. This may be attributed to an already high tumor burden from the large number of transplanted tumor cells, where direct iNKT effector functions may be quickly overwhelmed. Interestingly, while very few iNKT cells remained in the tumor microenvironment at the time of tumor harvest, the majority of iNKT cells that remained were CD4^−^/CD8^−^ double negative (DN) (data not shown), indicative of increased cytotoxic ability [58]. Recent studies performed in the murine prostate TRAMP model demonstrated that CD1d-positive TRAMP prostate tumor cells could induce cytokine defects in tumor-infiltrating DN iNKT cells [59]. CD1d-expressing cancer cells in the primary breast tumor may provoke similar defects in iNKT function. Alternatively, as TM40D tumor cells were able to metastasize to lung in a CD1d-dependent manner, it is possible that the direct cytotoxic functions of DN iNKT cells may be more important in targeting circulating tumor cells, rather than decreasing primary tumor burden. Further studies are required to more fully characterize the direct vs. indirect cytotoxic functions of iNKT cells in preventing spontaneous breast cancer metastasis.

In order to assess the relevance of these findings to human breast cancer, we analyzed the expression of CD1d in several human mammary cell lines of increasing metastatic potential by RT-PCR. We provide the first evidence that expression of CD1d in human mammary epithelial cells is lost in the transition from normal to invasive breast cancer. However, not all metastatic cell lines lost CD1d, as evidenced by the highly metastatic MDA-MB-468 cells. Interestingly, these cells were originally acquired from an African-American patient, while the equally metastatic MDA-MB-231 cells were acquired from a Caucasian patient [60]. Differences in genetic variation of CD1d haplotypes between these ethnicities has been previously reported, and possible alternate slicing or expression of CD1d between ethnicities is possible [61]. These findings may have important clinical implications in tailoring individual NKT-based immunotherapies for the treatment of breast cancer. Treatment with an anti-CD1d blocking antibody may be effective for CD1d-deficient tumors, however CD1d-expressing tumors would likely be more responsive to iNKT-activating therapies.

Studies in multiple murine tumor models have demonstrated that type I (iNKT) and type II NKT cells have opposing regulatory roles, with type I NKT cells demonstrating potent antitumor immune responses, and type II NKT cells exhibiting mainly immunosuppressive functions [14]. Type I NKT dominant tumors, such as 4T1 mammary carcinoma and CT26 colon carcinoma, have been shown to grow similarly in CD1d KO (type I and II deficient) and Jα18 KO (type I deficient) mice as in wildtype, but demonstrate increased metastasis in Jα18 KO mice [37]. In these models, the presence of type I NKT cells is the dominant factor in preventing metastasis, regardless of the presence or absence of suppressive type II NKT cells. In contrast, type II NKT dominant tumor models, such as subcutaneous 15-12RM fibrosarcoma and CT26-L5 colon carcinoma, grow normally in Jα18 KO mice but do not grow at all in CD1d KO mice, highlighting the requirement of suppressive type II NKT cells in promoting tumor progression of these cells. Importantly, all of these tumor models are CD1d-deficient, and the role of CD1d-expressing tumors in promoting a type I vs. type II NKT immune response in non-hematopoietic tumors has not previously been investigated. In a murine model of B cell lymphoma, CD1d-expressing tumor cells demonstrated increased tumor progression in Jα18 KO mice as compared to CD1d-deficient tumor cells [51]. This was suggested to be due to the ability of CD1d-expressing tumor cells to recruit suppressive type II NKT cells in the absence of type I NKT cells. Also in this model, CD1d KO mice were highly resistant to tumor progression of both CD1d-expressing and CD1d-deficient tumor cells, suggesting a dominance of suppressive type II NKT cells.

In order to elucidate the specific roles of CD1d-restricted NKT cells in our model of breast cancer, we utilized CD1d KO (type I and type II NKT cell deficient), Jα18 KO (type I NKT cell deficient) and RAG2 KO (B and T cell deficient) mice to compare tumor growth and metastasis rates between our CD1d-lo and CD1d-hi tumor cells. Our TM40D (CD1d-hi) tumors grew at the same rate in all mouse strains, similar to the type I NKT dominant 4T1 mouse model [37]. However, while the CD1d-deficient 4T1-implanted CD1d KO mice exhibited fewer metastases and increased survival as compared to Jα18 KO mice, our CD1d-expressing TM40D tumor cells showed the opposite effect, with significantly increased metastasis in the CD1d KO mice as compared to Jα18 KO mice. Similarly, while the Smyth group was able to inhibit 4T1 metastatic growth by *in vivo* anti-CD1d antibody blockade, our TM40D cells demonstrated significantly increased metastasis upon *in vivo* antibody blockade. As antibody blockade of CD1d-expressing tumors would similarly abrogate direct type II NKT recruitment, our TM40D cells may not be able to recruit type II NKT cells, as suggested in the B cell lymphoma study [51]. Clearly, both tumor and systemic expression of CD1d are important for preventing metastasis of TM40D tumors. However, the specific contributions of type I and type II NKT cells in regulating these tumors, as compared to other adaptive immune cells, cannot be concluded at this time.

In order to directly assess the role of type I vs. type II NKT cells in regulating CD1d-deficient tumors in our tumor model, we implanted TM40D-MB (CD1d-lo) tumors in wildtype, CD1d KO, Jα18 KO and RAG2KO mice. Similar to TM40D, TM40D-MB tumors were highly metastatic in the RAG2 KO mice, demonstrating the importance of adaptive lymphocytes in regulating both TM40D and TM40D-MB tumors. As with other type II NKT dominant CD1d-deficient tumor models, TM40D-MB tumors did not grow at all in CD1d KO mice, yet were able to grow and metastasize in type I NKT-deficient Jα18 KO mice. These findings further demonstrate the importance of suppressive type II NKT cells in promoting tumor progression of tumors with downregulated CD1d.

Preliminary experiments in our lab with Jα18 KO mice implanted with TM40D-MB tumors demonstrated a significant increase in splenic CD49b^+^ CD4^+^ TCRβ^+^ cells, believed to be type II NKT cells (unpublished results). This suggests that while CD1d expression in these tumor cells is downregulated, they may still be capable of being recognized by type II NKT cells. One possible explanation for the discrepancy in recognition of TM40D and TM40D-MB tumors by type II NKT cells is that it may not depend solely on the expression level of CD1d. Rather, the ability of CD1d-expressing tumor cells to promote type I over type II NKT immune responses may depend more on the type of tumor antigen presented by CD1d molecules. In support of this theory, a recent study demonstrated that inflammation-associated lipids presented by APCs in the tumor microenvironment preferentially recruit suppressive type II NKT cells [62]. In addition, glycolipid antigens recognized by type II NKT cells may differ from type I NKT cells, in that type II NKT activation does not require CD1d trafficking through endosomes [63], [64]. As a tumor progresses, the tumor microenvironment is well known to induce inflammation, which facilitates tumor growth and eventually fosters the recruitment of suppressive immune cells that further promote metastatic tumor progression [65]. It may be that downregulation of CD1d by the tumor is a fairly early event in tumor progression, and thus not associated with the late-stage inflammatory tumor microenvironment that preferentially recruits type II NKT cells. Thus, tumor downregulation of CD1d may be advantageous for evasion of iNKT-mediated immune surveillance in the early stages of tumor progression, when antigens presented by tumor cells such as TM40D may preferentially elicit type I over type II NKT antitumor immune responses. In late-stage metastatic breast cancer, such as in TM40D-MB, presentation of inflammatory tumor antigens by even a low level of CD1d may be sufficient to drive type II NKT-mediated immune suppression. In our tumor model, TM40D-MB tumors have been shown to be more metastatic to lung and significantly more metastatic to bone than TM40D tumors [8]. While the focus of this study was on early spontaneous metastasis to lung, future studies characterizing the ability of TM40D-MB cells in bone to preferentially recruit type II NKT cells may shed light on mechanisms that increase their bone metastatic potential.

In summary, the eradication of breast cancer through bolstering iNKT-mediated antitumor immunity remains a promising therapeutic direction. The results of this study further support the development of immunotherapeutics that increase the activation and function of type I iNKT cells, while inhibiting the suppressive effects of type II NKT cells. Based on our findings of the tumor-promoting effects of anti-CD1d monoclonal antibody treatment of CD1d-expressing tumor cells, current therapeutic strategies based on globally inhibiting CD1d by antibody blockade are not recommended. Rather, the CD1d expression status of the tumor and the stage of disease progression may be important considerations in tailoring future breast cancer immunotherapies that effectively promote iNKT-mediated antitumor immunity.

## Supporting Information

Figure S1 **Downregulation of the gene encoding CD1d in highly metastatic tumor cells identified by microarray.** Hierarchical cluster diagram of 86 genes (represented by 86 probe sets) that were over- and under-expressed in cells highly metastatic to bone (TM40D-MB) compared to TM40D cells. The Affymetrix probe set number, fold differences, *P*-value and identities of the genes are indicated. Data were analyzed by the Gene-Spring 5.0.3 array data analysis software (Silicon Genetics, Redwood City, CA) and were normalized in the dChip software (Harvard School of Public Health and Dana-Farber Cancer Institute, Boston, MA).
(TIF)
