# Supplementary figures and images for: CD1d-Expressing Breast Cancer Cells Modulate NKT Cell-Mediated Antitumor Immunity in a Murine Model of Breast Cancer Metastasis
Source: PLoS One. 2011 Jun 13;6(6):e20702. doi: 10.1371/journal.pone.0020702 (PMC3113806; doi:10.1371/journal.pone.0020702)

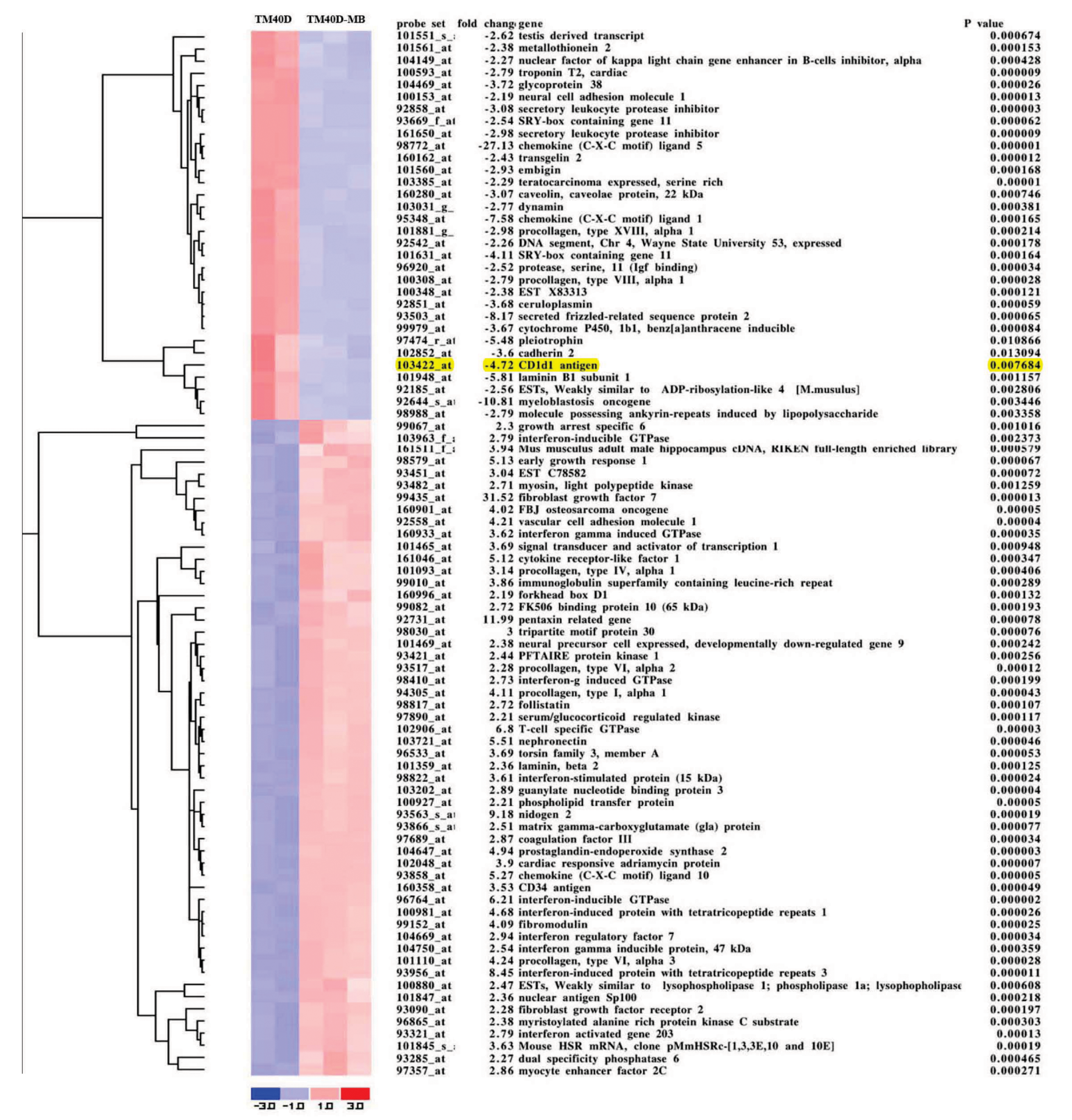

Supplement: Figure S1 — Downregulation of the gene encoding CD1d in highly metastatic tumor cells identified by microarray. Hierarchical cluster diagram of 86 genes (represented by 86 probe sets) that were over- and under-expressed in cells highly metastatic to bone (TM40D-MB) compared to TM40D cells. The Affymetrix probe set number, fold differences, P-value and identities of the genes are indicated. Data were analyzed by the Gene-Spring 5.0.3 array data analysis software (Silicon Genetics, Redwood City, CA) and were normalized in the dChip software (Harvard School of Public Health and Dana-Farber Cancer Institute, Boston, MA). (TIF) [file pone.0020702.s001.tif]
